# Hydrodissection technique with t‐tube placement in atelectatic ear

**DOI:** 10.1002/ccr3.3242

**Published:** 2020-08-25

**Authors:** Eleana Tzoi, Konstantinos Garefis, Anastasia Kupriotou, Vasileios Nikolaidis, Konstantinos Markou

**Affiliations:** ^1^ 2nd Academic Department of ENT Aristotle University of Thessaloniki Papageorgiou general hospital Thessaloniki Greece; ^2^ Department of Otolaryngology‐Head and Neck Surgery 2nd Academic Department of ENT Aristotle University of Thessaloniki Papageorgiou General Hospital Thessaloniki Greece

**Keywords:** atelectasis, chronic otitis media, tympanoplasty, ventilation tube

## Abstract

Hydrodissection technique is a safe way to establish a fully functional tympanic membrane in cases of early stages of atelectatic ears.

## Graphical abstract

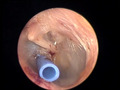

## 1 INTRODUCTION

The existence of a classification system is useful in documenting and following the atelectatic ear and is helpful with determining management. Often, nasal steroids and decongestants are sufficient. On failure, surgical management of the atelectatic ear is brought to the fore. It includes reconstruction of tympanic membrane either by perichondrium/cartilage/temporal fascia graft or by laser technique, with or without ventilation tube insertion. We present a case of atelectatic ear that was treated using hydrodissection technique and t‐tube insertion with good anatomical and functional results.

## 2 CASE REPORT

**Figure 1:**
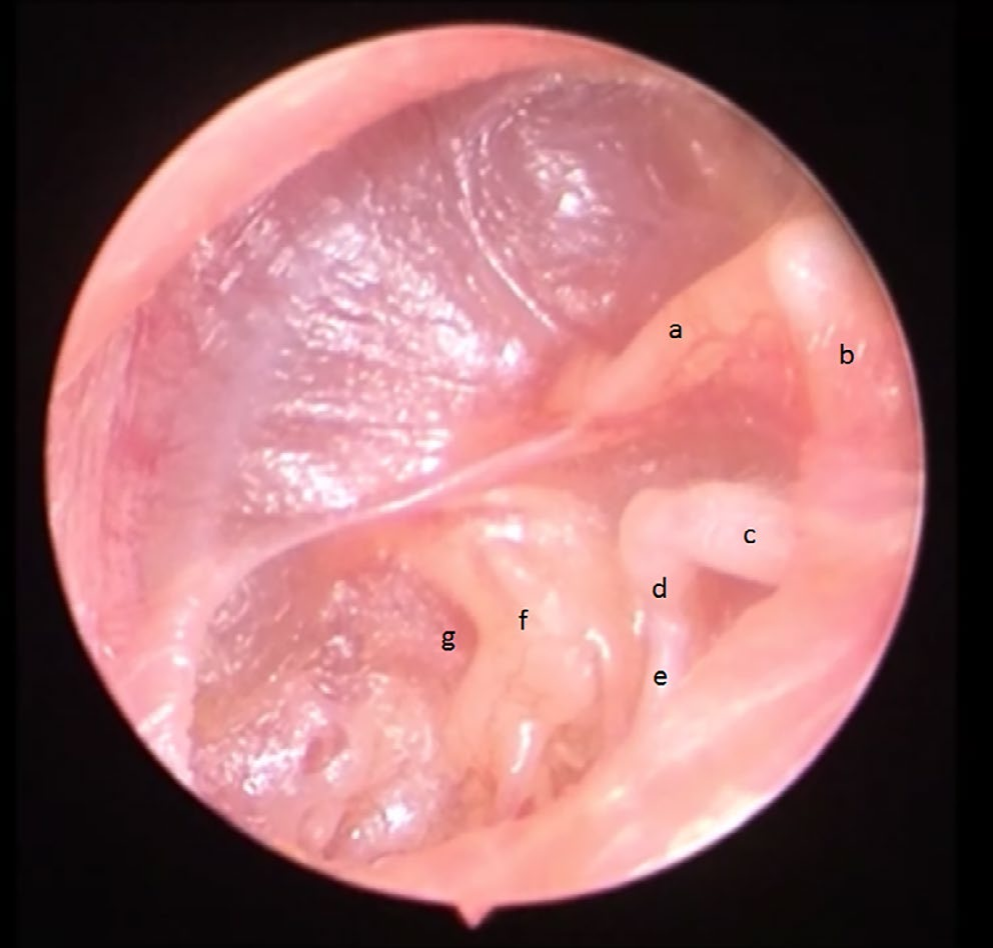
Left ear with completely retracted tympanic membrane. TM is adhered to the ossicles and promontory. a. malleus handle, b. Short process of malleus, c. long process of incus, d. incudostapedial joint and head of stapes, e. stapedius muscle, f. promontory, g. round window membrane

**Figure 2:**
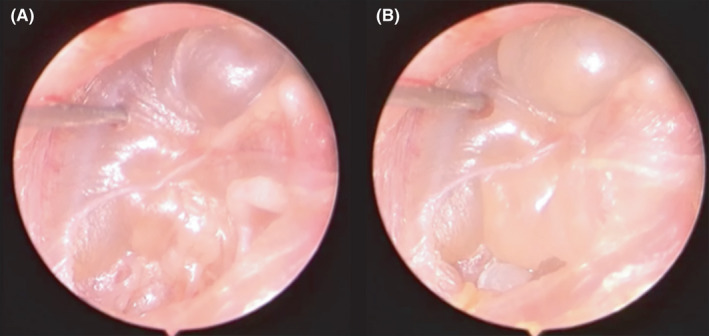
A and B, Injection of saline in the middle ear and complete detachment of tympanic membrane from the ossicular chain and promontory

**Figure 3:**
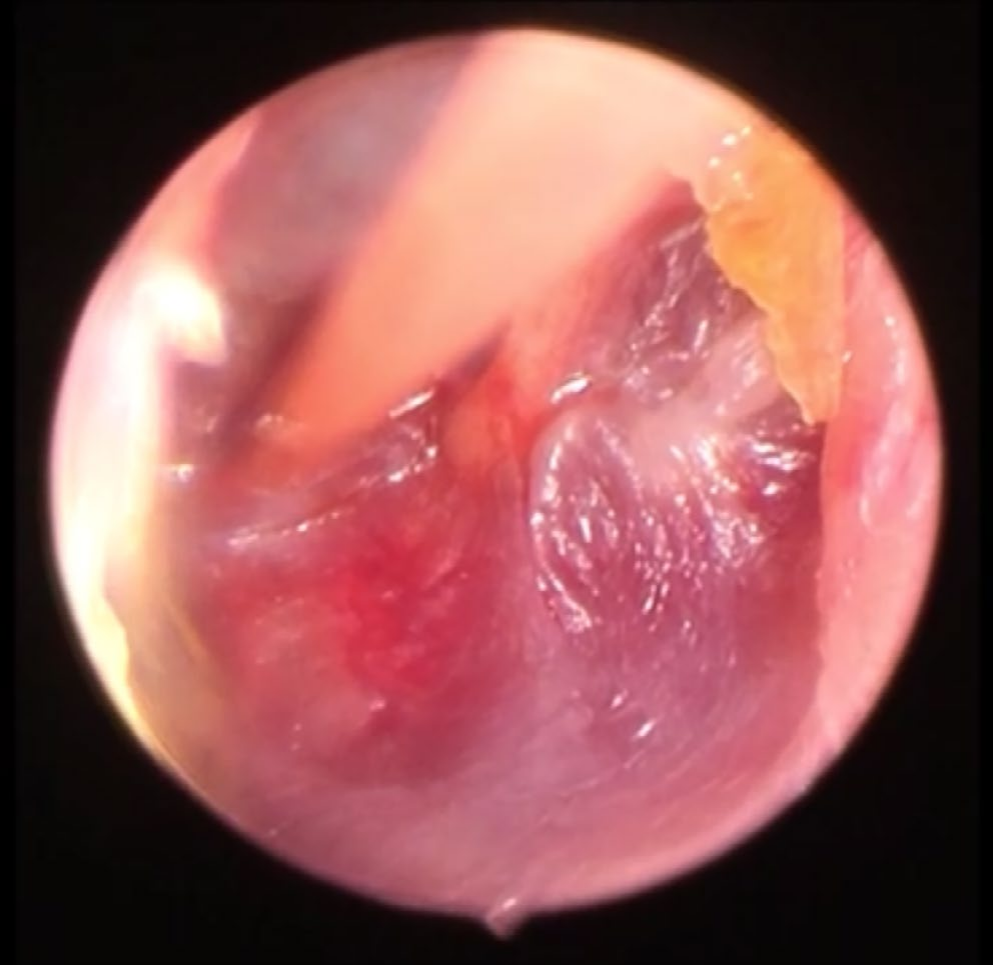
Myringotomy on the anterior‐inferior part of the TM and mucoblenoid fluid abstraction from the middle cavity

**Figure 4:**
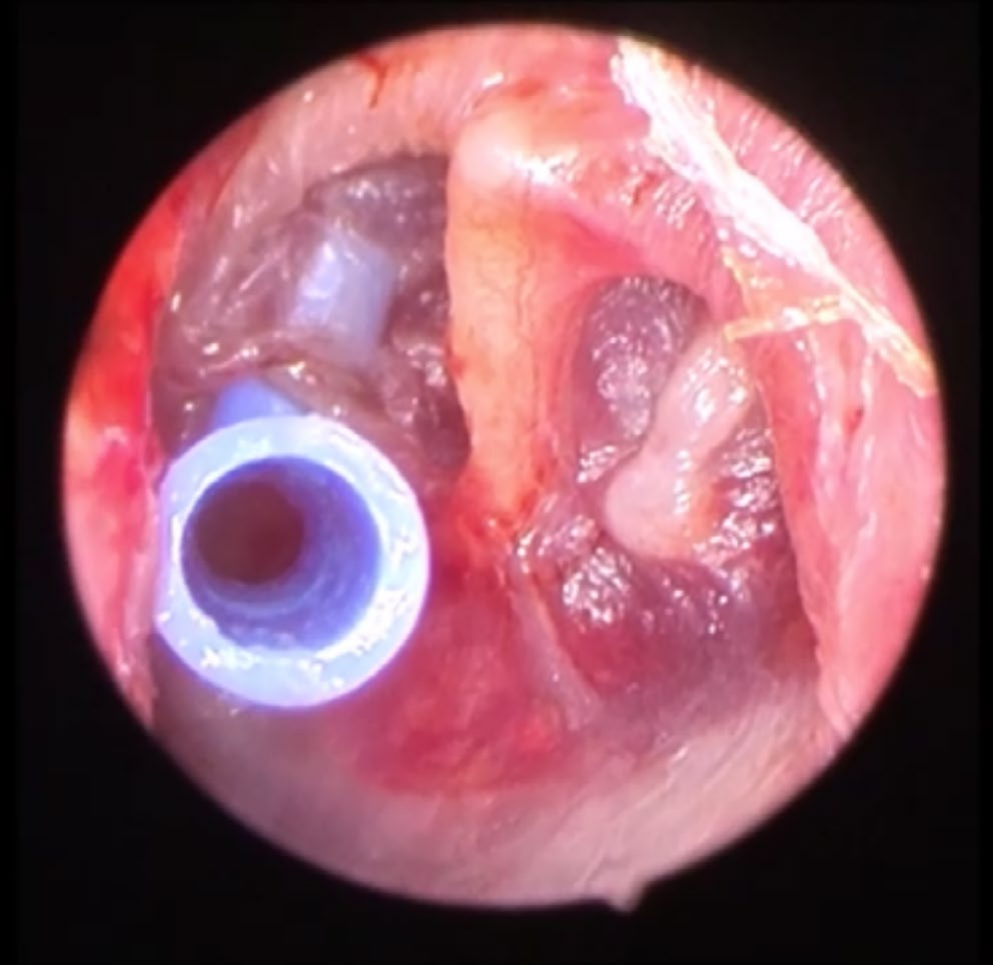
Concominant intraoperative ventilation t‐tube insertion

A 4‐year‐old female presented to the ENT clinic with bilateral hearing loss and ear fullness, progressively worsening over the past 3 weeks. She denied otorrhea, otalgia, tinnitus, vertigo, and headaches; she had no history of ear surgery, ear trauma, or significant noise exposure. Otoscopy revealed a diffused and retracted tympanic membrane involving both pars flaccida and pars tensa, progressing inward and touching the promontory (Grade IV in Sade's classification) (Figure 1). Fiber‐optic endoscopic evaluation revealed no sinus or nasopharynx inflammation. An audiogram was obtained and revealed conductive hearing loss and an air‐bone gap of 40 dB. She received medical treatment with nasal steroids, normal saline irrigations, and decongestants without improvement. Surgery was indicated, and hydrodissection technique took place. Endoscopically assisted, intratympanic injection of saline in the middle ear was performed, via trascanal approach, under general anesthesia. The drum was detached from the promontory and ossicular chain (Figure 2). Τhrough myringotomy on the anterior‐inferior part of the par tensa, mucoblenoid fluid (Figure 3) was withdrawn and a t‐tube was inserted (Figure 4). The procedure took place bilaterally.

**Figure 5:**
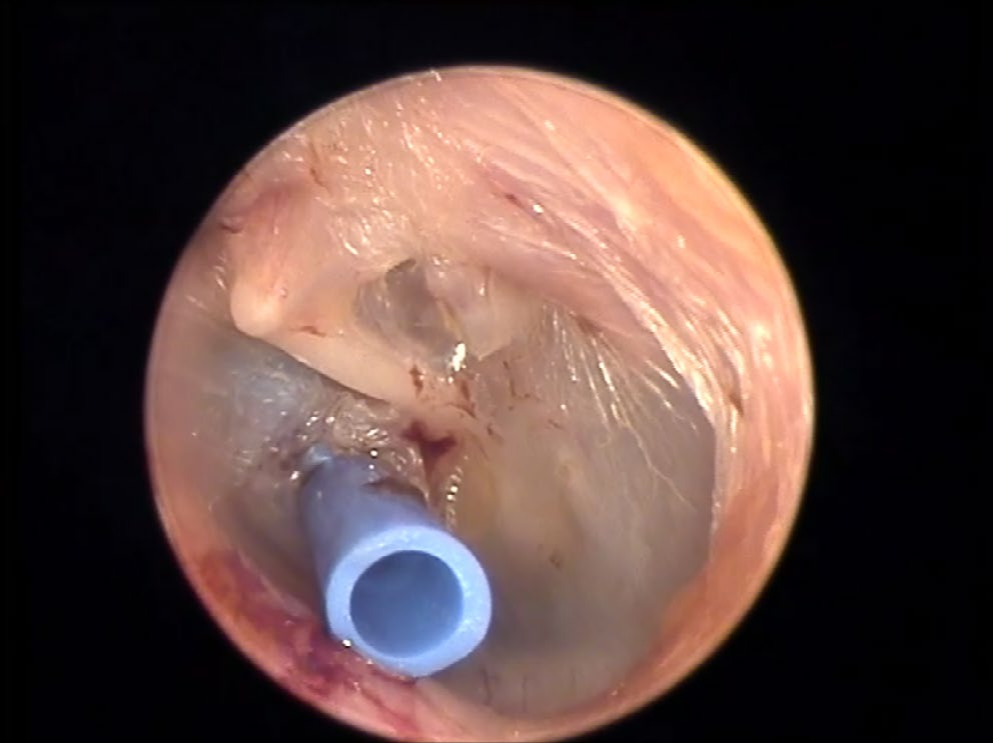
Postoperative otoscopic image with complete compensation of the atelectatic and atrophic tympanic membrane

Ten days postoperatively, audiogram showed comprehensive restoration of hearing. Otoscopy revealed complete compensation of the atelectatic and atrophic tymbanic membrane which was highly fuctional (Figure 5).

## 3 DISCUSSION

The atelectatic tympanic membrane poses a significant management challenge. The under‐ventilated middle ear and retracted drum can progress to ossicular erosion, progressive hearing loss, or cholesteatoma formation.^1^, ^2^ Management of this problem remains difficult given that the etiology of the atelectatic membrane is not completely understood. It is unknown whether Eustachian tube dysfunction and negative pressure in the middle ear are the only responsible for this problem.^3^ In‐ear instillation of traditional products, inflammation of upper respiratory tract, such as rhinitis and nasopharyngitis, craniofacial anomalies, immunosuppression, atopy, and low socioeconomic level are some of the contributing factors that increase the risk of transition to chronicity.^4^ The controversy is augmented by the fact that, early in the course of the disease, and even in the presence of incus necrosis, hearing loss is frequently minimal, and patients are mostly asymptomatic.^4^

A grading system that is a modification of Sades has been used to develop a treatment algorithm for this condition.^2^ The treatment of COM is medicosurgical. Surgery is a modality when medical treatment is insufficient, particularly in grade III‐IV.^2^ Surgical procedures consist of traditional tympanoplasty via cartilage/perichondrium graft, CO_2_ laser tympanoplasty and mastoidectomy in case of cholosteatoma.^5^ It may be accompanied by ossicular reconstruction and usually ventilation tube insertion preoperatively.^5^ The aim is to reverse atelectasis and to repneumatize the middle ear. In certain situations, such as the atelectatic ear, cholesteatoma, and revision tympanoplasty, temporal fascia and perichondrium have been shown to undergo atrophy and subsequent failure in the postoperative period, regardless of placement technique.^5^ The interest leads to the establishment of cartilage in TM reconstruction. Its rigid quality tends to resist resorption and retraction, even in patients with pervasive Eustachian tube dysfunction.^5^ Hydrodissection technique aims to re‐establish the tympanic membrane and repair middle ear pathology without impairment of the physical tympanoossicular chain. It includes normal anatomical and physiological reconstruction of the middle ear, is cost‐effective, easy to perform, no time‐consuming, bears no risk of disease transmission, nor risk of graft surveillance, and graft dissolution. It constitutes of an alternative endoscopic technique especially preferable in the pediatric population. Moreover, in adults it can be made in an outpatient basis, under topical anesthesia.

## 4 CONCLUSION

Hydrodissection technique is safe technique for tympanic membrane restoration that may lead to a more effective and faster surgical management of the early atelectatic cases without the existence of cholesteatoma. It appears to offer an extremely reliable method for reconstruction of the tympanic membrane in cases of atelectatic membrane with a significant improvement of hearing and no sign of recurrence.

## CONFLICT OF INTEREST

None declared.

## AUTHOR CONTRIBUTIONS

Eleana Tzoi: contributed to writing—original draft. Konstantinos Garefis: contributed to project administration. Anastasia Kupriotou: contributed to project administration, visualization. Vasileios Nikolaidis: contributed to conceptualization, investigation, and writing—review. Konstantinos Markou: contributed to supervision.
